# Association between Elevated Plasma Vitamin B12 and Short-Term Mortality in Elderly Patients Hospitalized in an Internal Medicine Unit

**DOI:** 10.1155/2023/6652671

**Published:** 2023-12-18

**Authors:** Benjamin Eduin, Camille Roubille, Stéphanie Badiou, Jean Paul Cristol, Pierre Fesler

**Affiliations:** ^1^Department of Internal Medicine, University Hospital of Montpellier, Montpellier, France; ^2^PhyMedExp, University of Montpellier, INSERM, CNRS, University Hospital of Montpellier, Montpellier, France; ^3^Department of Biochemistry and Hormonology, University Hospital of Montpellier, Montpellier, France

## Abstract

**Background:**

The prognostic value of vitamin B12 blood levels remains controversial. An association between elevated vitamin B12 and mortality has been reported, particularly among elderly patients with cancers and liver or blood diseases. The present study explored the relationship between mortality and elevated vitamin B12 levels in a population of unscheduled inpatients in an internal medicine unit.

**Methods:**

This retrospective observational analysis was conducted between August 2014 and December 2018. We compared 165 patients with elevated plasma vitamin B12 levels (>600 pmol/l) with a random sample of 165 patients with normal B12 levels who were hospitalized during the same period. Demographic, clinical, and biological characteristics were assessed during hospitalization. The primary endpoint was all-cause death at 1 year.

**Results:**

Patients with elevated B12 were younger, with a lower body mass index and lower plasma albumin than those with normal B12 (75 ± 16 years vs 79 ± 13 years, *p* = 0.047; 23 ± 5 vs 26 ± 7 kg/m^2^, *p*  <  0.001; and 33 ± 5 vs 35 ± 5 g/l, *p*  <  0.001, respectively). The prevalence of auto-immune disease and referral from an intensive care unit was higher among patients with elevated B12 (11% vs 5%, *p* = 0.043 and 36% vs 10%, *p*  <  0.001, respectively). After 1 year of follow-up, 64 (39%) patients with elevated B12 had died compared to 43 (26%) patients with normal B12 (*p* = 0.018). Multivariate analysis using the Cox proportional hazards regression model adjusted for age, gender, body mass index, intensive care unit hospitalization, albumin level, and the presence of solid cancer or autoimmune disease revealed elevated B12 to be associated with a significant risk of death in the first year of follow-up (hazard ratio: 1.71 [1.08–2.7], *p* = 0.022).

**Conclusion:**

Elevated B12 is an early warning indicator of increased short-term mortality, such as independently of age, cancer, or comorbidities, in patients hospitalized in an internal medicine department.

## 1. Introduction

Vitamin B12 (cobalamin) is an essential nutrient for humans and animals. The structure of the molecule and physiological functions are complex; however, the effects of B12 deficiency in humans were recognized long before its discovery, which gave rise to the name “antipernicious anemia factor.” The mechanism of vitamin B12 biosynthesis by microorganisms was only completely elucidated in 2007 [1].

As an essential nutrient, cobalamin must be obtained from food sources and is mainly stored in the liver. Ingested B12 is complexed with proteins, which are cleaved in the stomach by pepsin. Vitamin B12 is subsequently binded to intrinsic factor (IF), which is produced by stomach parietal cells. The B12/IF complex is absorbed in the terminal part of small intestine, in an absorption process which requires the “cubam” receptor, composed of cubilin (gp280) and amnionless proteins [2]. This receptor is also found in the proximal convoluted tubule of the kidney where it is involved in protein reabsorption [3, 4]. Imerslund–Grasbeck syndrome is characterized by megaloblastic anemia and proteinuria and is caused by reduced vitamin B12 absorption due to a cubilin mutation [5]. After absorption, cobalamin binds to a transport protein named transcobalamin, which is secreted by the vascular endothelium and enables the molecule to enter the cell via the TCblR/CD320 receptor [6].

Cobalamin has a role as a cofactor in two enzymatic reactions which are the regeneration of methionine from homocysteine by methionine synthase and the isomerization of methylmalonyl-CoA to succinyl-CoA by methylmalonyl-CoA mutase; succinyl-CoA enters then in the tricarboxylic acid cycle [7–9]. These roles explain certain consequences of B12 deficiency such as megaloblastic anemia and neurological disorders [10].

Elevated serum or plasma vitamin B12 level is a common abnormality that is often observed in patients with solid neoplasms, blood cancers, myelodysplasia, alcoholism, and liver disease [11, 12]. However, the majority of patients with high B12 levels have no associated pathological conditions, leaving the causal link uncertain. Elevated B12 has been reported to be associated with an increased risk of cardiovascular mortality [13].

The present study aimed to evaluate the association between elevated vitamin B12 and mortality in patients admitted to an internal medicine department and to identify common characteristics of patients with elevated B12.

## 2. Methods

### 2.1. Study Design and Population

This is a monocentric retrospective study of patients who were consecutively admitted to our internal medicine department between August 2014 and December 2018. This period was chosen in order to analyze B12 levels over a minimum of 3 years of follow-up using the same biochemistry analyzer (Roche-Cobas©, Electrochimiluminescence assay) implemented in our biochemistry department in August 2014.

During the study period, 791 participants underwent vitamin B12 testing with a mean plasma B12 concentration of 444 ± 332 pmol/L. Of these, 49 (6.2%) had low B12 (<150 pmol/l), 165 (21%) had elevated B12 (>600 pmol/l) and 577 (73%) had normal B12.

To compare patients with elevated B12 with those with normal B12, we randomly selected 165 from the 577 patients with normal B12. We divided the inclusion period into 6-month intervals. Within each interval, we randomly selected 18-19 patients using the Excel random number generator (Microsoft Corporation, 2016).

### 2.2. Study Outcomes

Demographic and clinical characteristics including age, gender, weight, and body mass index (BMI) were extracted from medical records of admission. Conditions known to be responsible for high B12 levels such as liver disease, blood cancer, and alcoholism were assessed, as well as comorbidities including chronic kidney disease, chronic heart failure, chronic lung disease, dementia, solid cancers, and diabetes. Comorbidities were defined as “present” or “absent” at the time of admission. We have identified treatments, including the use of metformin or proton pump inhibitors (PPI), that are known to lower B12 levels [14–17].

We have collected some biological characteristics including hemoglobin, mean corpuscular volume, vitamin B9, ferritin, transferrin saturation coefficient, albumin, creatinine, and C-reactive protein at admission. Renal function was assessed by estimated glomerular filtration rate (eGFR) using the CKD-EPI equation (ml/min/1.73 m^2^).

The primary endpoint was all-cause death at 12 months, based on data from the national database [18]. Secondary outcomes were all-cause death at 1 and 3 months. This study and all of its protocols were approved by the Institutional Review Board of Montpellier University Hospital (IRB-MTP_2022_06_202201142).

### 2.3. Statistical Analyses

Continuous variables are expressed as mean±standard deviation (SD) or median and interquartile range in the case of skewed distribution. Variables were compared using Student's *t*-test preceded by log-transformation where appropriate. Categorical variables are expressed as percentages and were compared using Fisher's exact test. Survival analysis was performed using Kaplan–Meier curves and the log-rank test. Multivariate analysis was performed using the Cox proportional hazards regression model (expressed as hazard ratio (HR) with corresponding *p* values). We compared mortality between groups after adjusting for factors that showed significant differences in univariate analysis including gender. Statistical analyses were performed using IBM SPSS Statistics 25 software. *p* values of <0.05 were considered to be statistically significant.

## 3. Results

### 3.1. Study Population

The characteristics of the study population are shown in Table 1.

There were no significant differences in gender or reason for hospitalization between the normal and elevated B12 groups (Table 2). The mean age was significantly higher in the normal group than the elevated B12 group. Mean BMI and duration of hospitalization were significantly lower and higher, respectively, in the elevated B12 group, and referral from the intensive care unit (ICU) was more prevalent in the elevated B12 group.

Overall, the most common comorbidities were chronic heart failure, diabetes mellitus, chronic kidney failure, and dementia. Prevalence of each comorbidity was not statistically different between the two groups, except for auto-immune diseases, which were more common in the elevated B12 group. Notably, less than one-fifth of participants with elevated B12 had cancer or liver disease.

Hemoglobin, mean corpuscular volume, creatinine, and C-reactive protein levels were similar in the two groups. Vitamin B9 and ferritin levels were higher and albumin levels were lower in the elevated B12 group compared to the normal group.

Patients with normal B12 received more treatments, although there was no significant difference in the use of metformin or PPIs between groups.

### 3.2. Outcomes

#### 3.2.1. Univariate Analysis

**Figure 1:**
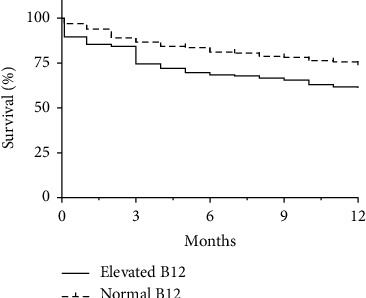
Kaplan–Meier survival curves during the first year comparing patients with normal B12 (dashed curve) with patients with elevated B12 (solid curve).

The overall median follow-up duration was 36 months. There was no significant difference in follow-up duration or mortality at the end of follow-up between groups (Table 3). The median time from admission to death was 6 months in the elevated B12 group and 20 months in the normal group (*p*  <  0.001). Kaplan–Meier analysis over 12 months confirmed that mortality during the first year of follow-up was significantly different between the two groups (Figure 1). Consequently, the mortality rates at 1, 3, and 12 months were significantly higher in the elevated B12 group (Table 3).

Overall, participants referred from the ICU showed similar Kaplan–Meier survival curves to those of other patients (log-rank *p* = 0.67). Moreover, participants with a solid cancer had a higher risk of short-term death, particularly at 1 year (61 vs 29%, *p*  <  0.001).

#### 3.2.2. Cox Multivariate Analysis

**Figure 2:**
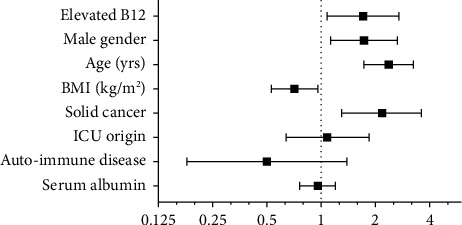
Forest plot illustrating the results of multivariate Cox analysis for death at 12 months. Hazard ratio with 95% confidence interval for presence vs absence (categorical variable) or per standard deviation (continuous variable); BMI = body mass index (kg/m^2^); ICU = intensive care unit.

The association between short-term (1-year) mortality and vitamin B12 level remained significant after adjustment for differences in age, gender (male), BMI, solid cancer, ICU origin, autoimmune diseases, and albumin level (HR 1.71 [1.08–2.70], *p* = 0.022; Figure 2). In multivariate analysis, other factors associated with 1-year mortality were age (HR 2.37 [1.73–3.25], *p*  <  0.001), gender (HR 1.73 [1.13–2.65], *p* = 0.012), BMI (HR 0.71 [0.53–0.96], *p* = 0.026), and solid cancer (HR 2.18 [1.30–3.60], *p* = 0.003).

## 4. Discussion

In our study, high B12 is associated with an increased risk of short-term all-cause mortality. We found that the 1-year prognosis was worse among patients with a B12 level >600 pmol/l, independent of various variables including significantly different baseline characteristics.

Our study population consisted mainly of elderly patients, which is typical of studies conducted in an internal medicine unit where patient are mainly referred from the emergency department [19]. The mean ages of the normal and elevated B12 groups suggest that there is no association between increased age and high B12 levels. This finding is supported by the results of studies with populations of different ages, such as older patients recruited from the internal medicine department in the study of Brah et al. [20], or rather younger patients recruited from the general population in the study of Flores-Guerrero [21]. It should be noted that the B12 levels measured in the present study correspond to those of these populations, suggesting the absence of geographical influence, at least in developed countries.

An interesting finding of the present study is the prevalence of high B12 in ICU-referred patients. A previous study of critical ill patients [22] showed that elevated B12 was independently associated with a poor 3-month prognosis. However, we did not find that patients referred from the ICU were at increased risk of death.

It has been suggested that cobalamin is released from the liver under critical conditions, and high B12 is considered a marker of liver injury [23, 24]. This is supported by our finding that patients with high B12 had higher ferritin and vitamin B9 levels than those with normal B12. Vitamin B9 is also stored in the liver, and the metabolism of vitamins B9 and B12 uses the same pathways [8]. Similarly, hyperferritinemia appears to be associated with liver dysfunction rather than inflammation, which is supported by our finding that C-reactive protein was similar in both groups. Corcoran et al. have previously found that B12 level is not related to inflammation [25].

We found that high B12 is associated with lower BMI and lower serum albumin. A recent study of elderly patients admitted for heart failure showed that undernutrition is associated with a poor prognosis [26], which was also found to be true for healthy elderly patients [27]. These findings suggest that vitamin B12 could be one of several markers of vulnerability and therefore a poor prognosis, especially in a frail population. Of note, we found no association between B12 levels and the most common comorbidities such as chronic heart failure, diabetes mellitus, chronic renal failure, or dementia.

High B12 has been reported to be associated with the presence of solid cancers as well as an increased short-term risk of cancer [28]. However, this is still controversial [29], and the present study found the prevalence of cancer was low in patients with elevated B12. It is possible that the persistence of high B12 levels over time leads to an increased risk of cancer, as recently suggested by Lacombe et al. [30]; however, there does not appear to be a link with any specific cancer, and liver cancers are not necessarily over-represented in the context of high B12 [31]. In the present study, blood diseases were found to be the least frequent comorbidity. This is surprising as hematological malignancies may be associated with elevated B12 [32], which may be a marker of high-risk myelodysplasia [33]. Of course, elderly patients may have unknown myelodysplastic conditions.

We found an association between short-term all-cause mortality and elevated B12, which is consistent with the current medical literature. Valdivia et al. [34] found that high B12 was significantly associated with an increased risk of death at 1 year in elderly patients admitted to an internal medicine department. However, Robinson et al. [35] did not observe a similar relationship. In the present study, this association remained significant after adjustment for cancer, BMI, albumin level, and ICU referral, suggesting B12 is an independent marker of poor prognosis. High B12 levels have been suggested as part of a frailty score in association with cancer, liver disease, or cardiovascular disease [36–38]. Indeed, if elevated B12 is independently associated with mortality, combining this with other poor prognostic factors such as low albumin or chronic disease is likely to improve its impact. Using this approach could lead to better management and outcomes for patients with high B12 and multiple comorbidities. However, it is not yet clear from the present study and from the literature whether a high B12 level is simply a marker of serious disease or whether it plays a direct role in increased mortality.

### 4.1. Limitations

Our study has several limitations that should be acknowledged. First, it is a retrospective analysis of a cohort of patients hospitalized in an internal medicine unit with a relatively small sample size. This may explain the fact that we did not identify an association between high B12 and cancer or liver disease, as patients with existing blood or liver diseases were more likely to be referred to other specialized departments. Secondly, the analysis of B12 level at inclusion only provides a snapshot of a patients' condition at a specific time. The median time to death was short in patients with high B12 (6 months), so the analysis of B12 levels at discharge and 2 months after hospitalization may clarify the influence of transient and persistent high B12 on prognosis. Thirdly, the mean difference in age between the groups was 4 years at inclusion. So, even though we have adjusted the analysis for age, a significant residual bias could remain, as mortality is strongly related to age.

## 5. Conclusion

Elevated B12 is an early warning indicator of increased short-term mortality, independently of age, gender, BMI, ICU admission, albumin level, and the presence of solid cancer or autoimmune disease in patients admitted to an internal medicine department. Although the mechanism behind this association remains unclear and warrants further research, our findings highlight the importance of close follow-up of elderly patients with elevated B12.

## Figures and Tables

**Table 1:** Population characteristics.

	Total, *n* = 330	Normal B12 (*n* = 165)	Elevated B12 (*n* = 165)	*p* value
Clinical				
Female	175 (53)	85 (51.5)	90 (54.5)	0.66
Age (years)	77 ± 15	79 ± 13	75 ± 16	**0.047**
Age of death (years)	84 ± 9	84 ± 9	83 ± 10	0.463
Weight (kg)	66 ± 18	68 ± 17	63 ± 16	**0.008**
BMI	25 ± 7	26 ± 7	23 ± 5	**<0.001**
From ICU	76 (23)	16 (10)	60 (36)	**<0.001**
Hospitalization duration (d)	20 ± 19	16 ± 15	23 ± 22	**<0.001**
Comorbidities				
Liver diseases	41 (12)	16 (10)	25 (15)	0.181
Chronic kidney diseases	106 (32)	59 (36)	47 (29)	0.195
Blood cancers	21 (6)	7 (4)	14 (8)	0.175
Solid cancers	36 (11)	13 (8)	23 (14)	0.111
Chronic heart failure	143 (43)	73 (44)	70 (42)	0.739
Chronic lung diseases	77 (23)	45 (27)	32 (19)	0.118
Autoimmune diseases	27 (8)	8 (5)	19 (11)	**0.043**
Diabetes mellitus	108 (33)	60 (36)	48 (29)	0.197
Dementia	86 (26)	50 (30)	36 (22)	0.103
Alcoholism	46 (14)	18 (11)	28 (17)	0.152
Biology				
Hemoglobin (g/dl)	11 ± 2	11 ± 2	11 ± 2	0.49
MCV (fl)	92 ± 8	92 ± 8	93 ± 9	0.36
B9 (nmol/l)	22 ± 21	18 ± 18	25 ± 23	**0.006**
B12 (pmol/l)	634 ± 490	301 ± 87	967 ± 502	**<0.001**
Creatinine (*μ*mol/l)	126 ± 97	134 ± 106	117 ± 87	0.122
eGFR (ml/min/1.73 m^2^)	57 ± 29	53 ± 27	61 ± 31	**0.018**
Albumin (g/l)	34 ± 5	35 ± 5	33 ± 5	**0.001**
Ferritin (median-IQR) (*μ*g/l)	286 [153–568]	215 [126–416]	404 [211–730]	**<0.001**
Transferrin saturation (%)	21 ± 20	19 ± 16	22 ± 23	0.132
CRP (median-IQR) (mg/l)	46 [13–119]	42 [11–118]	49 [17–124]	0.101
Treatments				
Numbers of treatment	7 ± 4	7 ± 3	6 ± 4	**0.003**
Proton pump inhibitors	149 (45)	78 (47)	71 (43)	0.507
Metformin	39 (12)	25 (15)	14 (9)	0.087

Data are shown as mean ± standard deviation, median (interquartile range), or *n* (percentage). BMI = body mass index, kg/m^2^; ICU = intensive care unit; MCV = mean corpuscular volume; eGFR = estimated glomerular filtration rate; CRP = C-reactive protein. Bold *p* values are < 0.05.

**Table 2:** Main causes of hospitalization.

	Total (%)	Elevated B12 (%)	Normal B12 (%)
Anemia	7 (2.1)	4 (2.4)	3 (1.8)
Alteration of general state	66 (20)	30 (18.2)	36 (21.8)
Dyspnea	18 (5.5)	10 (6.1)	8 (4.8)
Acute heart failure	42 (12.7)	18 (10.9)	24 (14.5)
Sepsis	111 (33.6)	59 (35.8)	52 (31.5)
Acute kidney failure	15 (4.5)	8 (4.8)	7 (4.2)
Hypertension	8 (2.4)	1 (0.6)	7 (4.2)

*p* chi^2^ = 0.95.

**Table 3:** Results of univariate analysis: death at 1, 3, and 12 months in patients with normal B12 levels compared with patients with elevated B12 levels.

	Total	Normal B12	Elevated B12	*p* value
Death at 1 month (%)	34 (10.3)	10 (6.1)	24 (14.5)	**0.009**
Death at 3 months (%)	64 (19.4)	22 (13.3)	42 (25.5)	**0.008**
Death at 12 months (%)	107 (32.4)	43 (26.1)	64 (38.8)	**0.018**
Death at end of follow up	206 (62.4)	106 (64.2)	100 (60.6)	0.57

Bold *p* values are < 0.05.

## Data Availability

The data used to support the findings of this study are available from the corresponding author upon reasonable request.
